# Critical Limb Ischemia in a Young Patient With a Mechanical Aortic Valve Leading to Bilateral Above-Knee Amputation

**DOI:** 10.7759/cureus.15046

**Published:** 2021-05-15

**Authors:** Shiwani Kamath, Abid Ulhaque, Rias Ali, Johnathan Frunzi

**Affiliations:** 1 Department of Internal Medicine, Medical Center of Trinity, Trinity, USA; 2 Department of Radiology, Medical Center of Trinity, Trinity, USA; 3 Department of Cardiology, Medical Center of Trinity, Trinity, USA

**Keywords:** mechanical aortic valve complications, bilateral thrombosis, anti-coagulation, critical limb ischemia, non-compliance

## Abstract

This case reports a 24-year-old female with prior aortic insufficiency who underwent a mechanical aortic valve replacement with subsequent transient ischemic attacks and documented subtherapeutic international normalized ratio (INR). She presented with pain and bilateral lower extremity pulselessness. Workup showed a supratherapeutic INR, no thrombus on echocardiogram, and computed tomography angiography demonstrating no flow in the bilateral common femoral arteries. Patient failed repeated thrombectomies and had a bilateral above-knee amputation. The patient was suspected to have acute on chronic emboli from the mechanical aortic valve and further testing did not elucidate contributory causes of her critical limb ischemia. We believe this is the first documented case of bilateral lower extremity ischemia due to mechanical valve complications.

## Introduction

This article was previously presented as a poster at the 2020 American College of Physicians Florida Chapter Annual Scientific Meeting on December 4, 2020.

Mechanical heart valve replacements are offered for all four cardiac valves, either through traditional open heart surgery or via minimally invasive methods, with a total of nearly 400,000 valves being replaced each year [1]. Two types of heart valves are available, mechanical and bioprosthetic [2]. Of the heart valves, aortic valves are the most commonly replaced, usually for aortic valve stenosis, but other causes can include aortic regurgitation and bicuspid aortic valves [3-5]. The aortic valve insertion systems offered include surgical aortic valve repair and transcatheter aortic valve replacement [6].

Mechanical valve replacement surgery has a low intra-operative mortality rate averaging around 2-3% [7]. Intra-operative complications include vascular complications, paravalvular leak, and conduction abnormalities [6]. Post-operative complications include primary valve failure and dehiscence, thromboembolism, mechanical obstruction by pannus, endocarditis, hemolysis, and hemorrhage [1]. Patients often achieved low mortality within 30 years of operation with survival averaging 83-92% across valve and event types [7].

Transthoracic echocardiograms (TTE) are the test of choice for serial monitoring of patients with mechanical and bioprosthetic heart valves [2]. Initial screening should occur after valve replacement to establish a baseline [8]. Currently, guidelines recommend screening only bioprosthetic valves 10 years after or in the presence of symptoms signifying valve-related problems, and yearly thereafter [9]. The American College of Cardiology recommends lifelong Vitamin K antagonists for mechanical heart valves and Vitamin K antagonists three months after surgery for bioprosthetic heart valves [9].

Here, we present the case of a patient who underwent a mechanical heart valve replacement and thereafter developed emboli leading to critical limb ischemia.

## Case presentation

**Figure 1:**
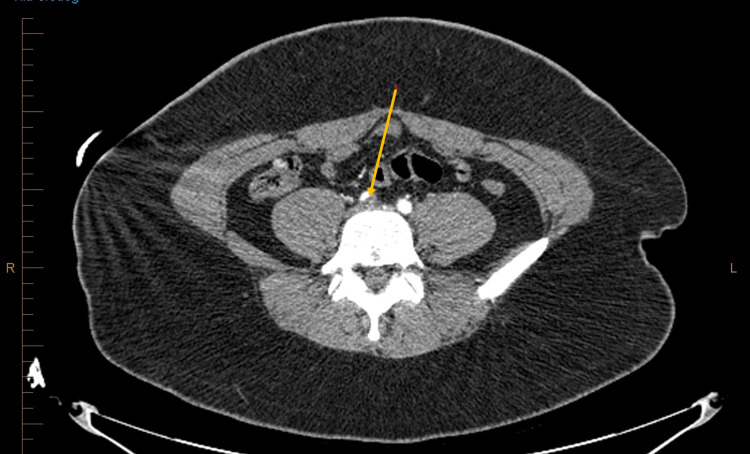
Orange arrow showing thrombus in the right common iliac artery.

**Figure 2:**
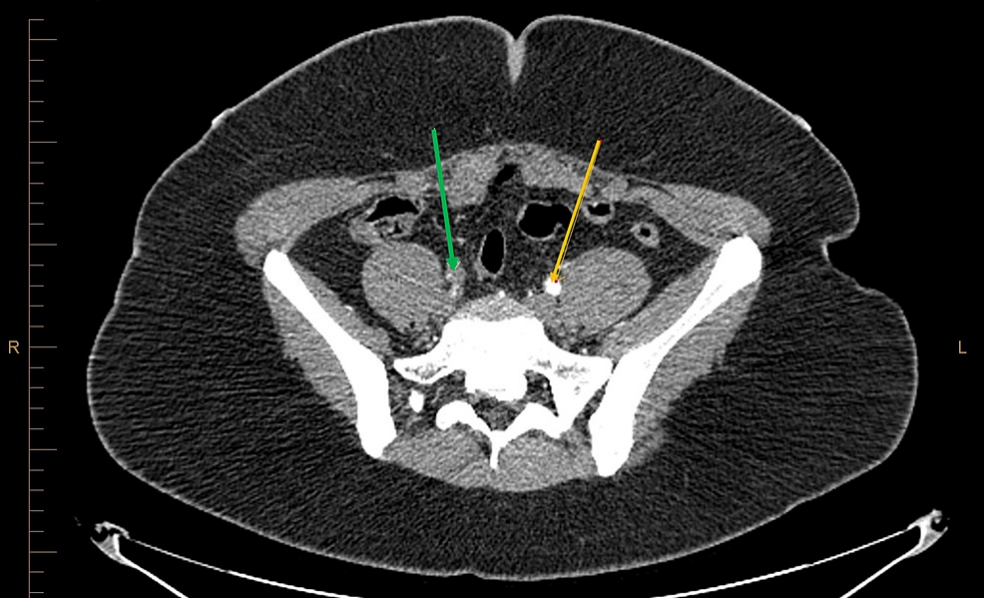
Green arrow showing complete occlusion of the right external illiac artery compared to orange artery showing patent left external iliac artery.

**Figure 3:**
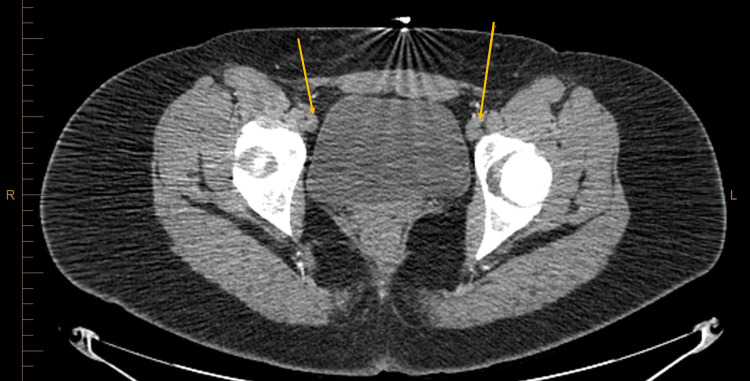
Complete occlusion of bilateral femoral arteries.

**Figure 4:**
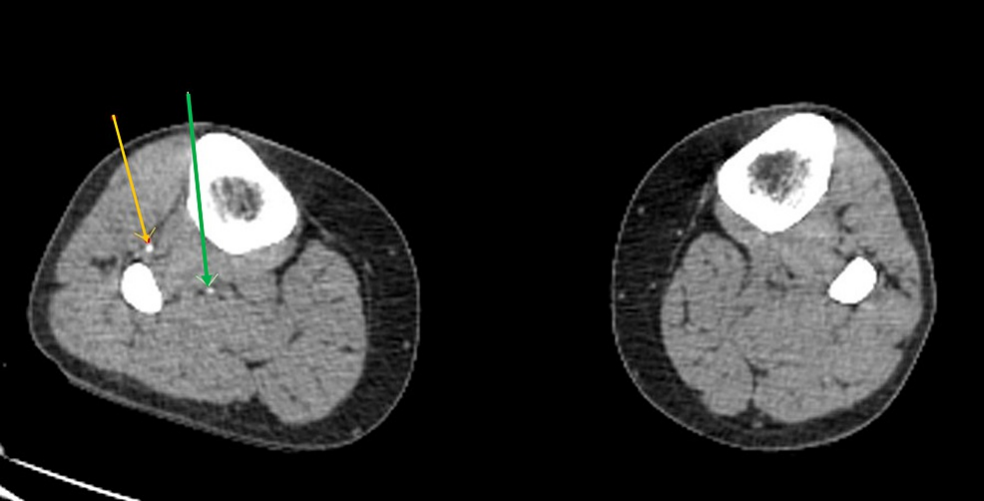
The green and orange arrows on the right showing contrast and blood flow in the right popliteal and superficial saphenous artery, respectively, albeit very small in caliber. No runoff contrast is seen throughout the left lower extremity.

Our patient is a 24-year-old African American female with a past medical history of bicuspid aortic valve and aortic insufficiency with aortic valve replacement eight months prior with a 25 mm On-X mechanical prosthesis aortic valve on warfarin. Recent history was significant for two transient ischemic attacks (TIAs) due to medication non-compliance and previously documented subtherapeutic international normalized ratios (INRs). She presented to the emergency room with complaints of pain in both her lower extremities for nine months. The pain was gradually progressive, bilateral, ascending from the feet to the knees, burning in character, aggravated by touch and ambulation, and relieved by rest. She admitted to recent medication non-compliance, followed by taking higher doses of warfarin at onset of symptoms. On physical examination, the bilateral lower extremities were hyperpigmented, severely tender, and cold to touch. Pulses were non-palpable below the femoral arteries. INR was 6.17. TTE and transesophageal echocardiogram (TEE) showed no evidence of thrombus. Computed tomography angiography (CTA) of the abdomen/pelvis/lower extremity revealed multiple thrombi of distal vessels (Figures 1, 2) and very small caliber, thread-like bilateral sapheno-femoral arteries with no flow in the bilateral common femoral arteries (Figure 3) and distal vessels (Figure 4). Critical limb ischemia secondary to acute on chronic emboli from the mechanical heart valve was suspected with showering of emboli prior to cardiac imaging.

Catheter-assisted thrombolysis with bilateral aspiration thrombectomy was attempted with no clinical improvement. The lower extremity ischemia progressed and the patient underwent bilateral above-knee amputation. Although wound care and anticoagulation were continued, ischemia and necrosis of the right stump continued. Further laboratory testing for prothrombic states with protein C, protein S, lupus anticoagulant, antiphospholipid antibodies, and homocysteine levels were within normal limits. Repeat CTA showed stenosis of right common and external iliac arteries and superficial, common, and deep femoral arteries with extensive gas around the right femoral stump. Repeat catheterization failed to revascularize the right stump, and the patient was transferred to a tertiary care center for a right hip disarticulation.

## Discussion

Prosthetic valve thrombosis (PVT) is an unusual but severe complication of mechanical aortic valves. There is significant associated morbidity and mortality. Clinically significant PVTs occur at 0.3-1.3% per patient-year [10].

Our patient, within the first year of aortic valve replacement, reported non-compliance with anticoagulation treatment, which was later confirmed by multiple episodes of subtherapeutic INR during hospital visits for two episodes of TIAs. However, our patient presented with supratherapeutic INR and no TTE or TEE findings of valvular thrombi. This may be explained by patient-initiated self-treatment of possible thrombi, but does not explain the extensive thrombi present in bilateral lower limbs. The patient’s workup for common coagulopathies, such as lupus, protein C or S deficiency, diffuse inflammatory processes such as Takayasu’s arteritis, or anatomical defects predisposing her to thrombus, were all negative. During a literature review, no studies or cases of valve replacement resulting in limb ischemia were found. We believe this is the first identified critical limb ischemia as a complication of valve replacement. We must consider that our patient had an underlying predisposition for thrombogenicity that was not diagnosed during this admission. Further testing for antithrombin deficiency, Factor V Leiden, and Prothrombin gene mutation should occur.

This case also highlights the importance of valve selection. Traditionally, patients who are less than 55 years old, with no contraindication to warfarin, are favored to receive a mechanical valve. Other factors, such as high morbidity with reintervention, further favor placement of mechanical valves. Bioprosthetic valves have had less incidence of thrombogenesis, but usually have a shorter lifespan. In select cases, patients who develop thrombosis with mechanical valves have undergone subsequent bioprosthetic valve placement.

We propose that once our patient had her second TIA event with a subtherapuetic INR and persistent medication non-compliance, she should have been considered for a replacement with a bioprosthetic valve. Her thromboembolism risk would have decreased and she would not have had alternating sub and supratherapeutic INRs.

It remains unclear that the patient’s embolic event can be solely attributed to poor medication compliance and subtherapeutic INRs. Our investigation did not reveal any common underlying thrombotic disease. Finally, this case raises the importance of re-evaluating patients who are having difficulty maintaining a therapeutic INR with coumadin or who have had embolic events secondary to mechanical valves. Although patients with On-X valves are allowed a lower INR of 1.5-2.0 for improved cumulative endpoints of thombogenic and bleeding events [11], these valves have increased thrombogenicity overall [2].

## Conclusions

We discuss the first documented case in the literature of a patient who developed severe limb ischemia secondary to prosthetic mechanical valve thromboembolism. The patient did not have any of the common thrombotic diseases, no thrombus on an echocardiogram, and was supratherapeutic when she presented to the hospital with bilateral occluding emboli in her lower extremities. For patients with recurrent thromboembolic events with mechanical valves, especially in the context of poor compliance, consideration should be given to bioprosthetic valve replacement.
